# Impact of rectal gas evacuation on dosimetry and applicator displacement in cervical cancer brachytherapy

**DOI:** 10.1002/acm2.70490

**Published:** 2026-02-09

**Authors:** Haiyan Wu, Chengdian he, Mei Liu, Xiujuan Zhao

**Affiliations:** ^1^ Department of Gynecological Oncology Chongqing University Cancer Hospital Chongqing China

**Keywords:** applicator displacement, brachytherapy, dosimetry, rectal gas evacuation

## Abstract

**Objective:**

This study aimed to evaluate the impact of rectal gas evacuation on organ‐at‐risk (OAR) volumes, dose‐volume histogram (DVH) parameters, and applicator displacement during cervical cancer brachytherapy.

**Methods:**

Twenty‐one cervical cancer patients who received three‐dimensional brachytherapy at our center between November and December 2024 and presented with rectal gas were retrospectively included. Planning computed tomography (CT) images were acquired before and after rectal gas evacuation to evaluate changes in rectal and bladder volumes, as well as radiation dose variations to OARs (bladder, rectum, sigmoid, and small intestine). Dosimetric parameters analyzed comprised *D*_0.1cc_, *D*_1cc_, *D*_2cc_, and *D*_5cc_ (minimum doses delivered to the most irradiated 0.1, 1, 2, and 5 cm^3^ of the OARs, respectively), as well as *D*_max_ (maximum dose) and *D*_mean_ (mean dose). Displacements of the applicator tip and cervical stopper were quantified using a coordinate system based on pelvic bony landmarks.

**Results:**

Rectal volume decreased by 40.1% after gas evacuation, while bladder volume increased by 18.2%. *D*_0.1cc_, *D*_1cc_, *D*_2cc_, *D*_5cc_, and *D*_max_ in the rectum decreased significantly (*P* < 0.001) after gas evacuation, whereas no significant changes were observed in the DVH parameters of the other OARs. The mean displacements of the applicator tip and cervical stopper were 5.86 ± 3.64 mm and 4.23 ± 3.30 mm, respectively.

**Conclusions:**

Rectal gas evacuation results in a statistically significant and clinically relevant reduction in rectal volume and rectal dose, underscoring its importance as a routine clinical procedure. However, as it may induce millimeter‐level applicator displacement with clinically measurable dosimetric consequences, careful monitoring is warranted, with post‐evacuation replanning or, if necessary, applicator adjustment.

## 1 INTRODUCTION

Cervical cancer is the fourth most common malignancy among women worldwide. According to the statistical report released by the International Agency for Research on Cancer (IARC), nearly 660 000 women were newly diagnosed with cervical cancer, and cervical cancer caused approximately 350 000 deaths globally in 2022.^1^ Brachytherapy is a key component of definitive radiotherapy for patients with primary cervical cancer. It delivers a high dose of radiation directly to the primary tumor and surrounding high‐risk regions, resulting in a higher tumor control rate while sparing normal tissues.^2^, ^3^ However, complete sparing of adjacent organs is not always feasible, particularly for radiosensitive organs such as the rectum. Rectal dosimetric accuracy is highly influenced by anatomical variability. Volume changes in the bowels due to rectal gas are a major source of dosimetric uncertainty. Clinical data have shown that rectal gas is present in approximately 24% of patients with cervical cancer undergoing radiotherapy, resulting in increased rectal radiation exposure.^4^, ^5^ The National Comprehensive Cancer Network (NCCN) guidelines recommend maintaining rectal *D*_2cc_ below 65–75 Gy.^6^ Higher rectal *D*_2cc_ values are associated with an increased cumulative incidence of rectal treatment‐related events. Values exceeding 75 Gy are generally considered unacceptably high and may increase the risk of intestinal fistula.^7^, ^8^

Currently, two main clinical strategies are employed to minimize rectal radiation exposure: reducing rectal volume through enemas and increasing the anatomical space between the rectum and target area by hydrogel injection or placement of an adjustable balloon.^9^, ^10^, ^11^, ^12^, ^13^, ^14^ However, these conventional methods are technically complex and invasive, potentially increasing patient discomfort and the risk of procedure‐related complications. In this context, image‐guided monitoring and evacuation of rectal gas during brachytherapy may provide a practical and effective method to improve rectal dosimetry accuracy. Several preliminary studies have demonstrated that rectal gas evacuation can significantly reduce rectal radiation exposure, thereby lowering the risk of grade 2–4 rectal treatment‐related events.^15^, ^16^ However, these studies have limitations. They compared only the changes in rectal dose‐volume histogram (DVH) parameters before and after gas evacuation, without comprehensively evaluating concurrent changes in rectal and bladder volumes or the potential impact on applicator position.

Given these limitations, this study aimed to systematically assess changes in rectal and bladder volumes and corresponding DVH parameters before and after rectal gas evacuation to evaluate its impact on organs at risk (OARs). Additionally, the effect of gas evacuation on applicator displacement was investigated. These findings may inform the development of more precise and clinically feasible strategies for rectal protection in cervical cancer brachytherapy.

## 2 MATERIALS AND METHODS

### 2.1 Patient characteristics

**FIGURE 1:**
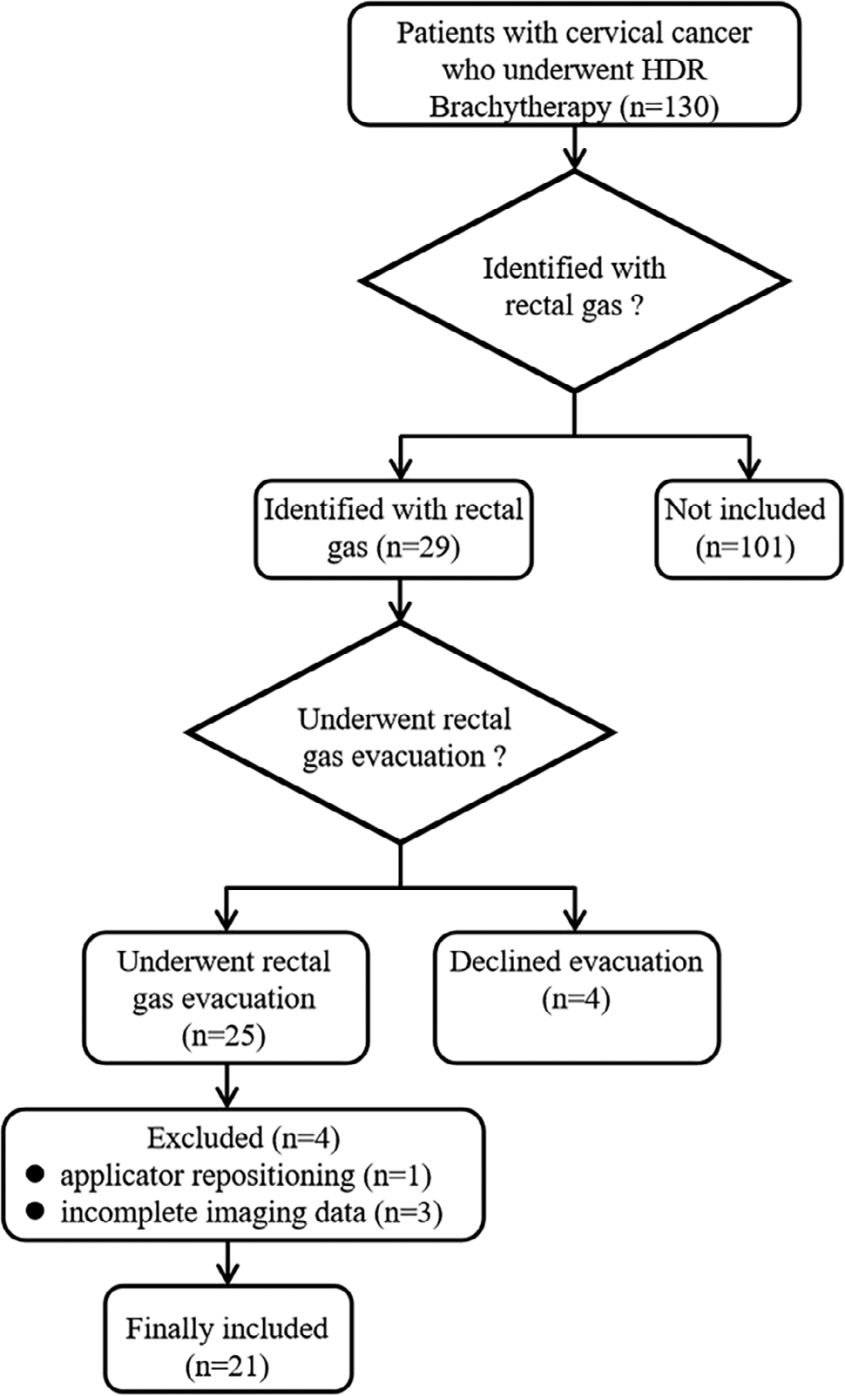
Flowchart illustrating patient selection.

This retrospective study was approved by the institutional review board (Approval ID: CZLS2023085‐A). The requirement for informed consent was waived by the institutional review board due to the retrospective nature of the study. All patient data were fully de‐identified prior to analysis, and the study was conducted in accordance with institutional data protection policies and the Declaration of Helsinki. Between November and December 2024, a total of 130 patients with cervical cancer underwent high‐dose‐rate (HDR) brachytherapy at our center. Rectal gas was identified in 29 patients. Of these 29 patients, 25 underwent rectal gas evacuation, whereas the remaining four declined the procedure due to personal preference (e.g., hemorrhoid‐related discomfort) or other clinical considerations. Four of the 25 patients were subsequently excluded: one due to applicator repositioning and three because of incomplete imaging data. Ultimately, 21 patients were included in the analysis (Figure 1). Among them, 17 patients underwent a single gas evacuation procedure and four patients underwent two procedures. Baseline patient characteristics are summarized in Table 1. All patients received external beam radiotherapy (EBRT) with a total dose of 45 Gy in 25 fractions, combined with concurrent weekly cisplatin monotherapy (40 mg/m^2^, 4–5 cycles). Brachytherapy was subsequently administered after EBRT at a prescription dose of 30 Gy delivered in five fractions, twice weekly over a period of 2.5 weeks.

**TABLE 1:** Patient and brachytherapy‐related characteristics.

Variables	Value
**Patient characteristics**	
Age, years (mean ± SD, range)	59.96 ± 9.75 (36–78)
Histological type	
Squamous cell carcinoma	18 (85.71%)
Adenocarcinoma	3 (14.29%)
FIGO stage (2018)	
IIA–IIB	11 (52.38%)
IIIA–IIIB	5 (23.81%)
IIIC1r–IIIC2p	5 (23.81%)
**Brachytherapy‐related characteristics**	
Number of brachytherapy fractions with rectal gas evacuation	
1–2	10 (40.00%)
3–4	7 (28.00%)
5–7	8 (32.00%)
Applicator type	
Tandem and ovoids	7 (28.00%)
Tandem with interstitial needles	12 (48.00%)
Tandem and ovoids combined with interstitial needles	2 (8.00%)
Vaginal cylinder	1 (4.00%)
Interstitial needles alone	3 (12.00%)

*Note*: Data are presented as *n* (%) unless otherwise specified. Patient characteristics are reported per patient (*n* = 21), whereas brachytherapy‐related characteristics are reported per brachytherapy fraction with rectal gas evacuation (*n* = 25). FIGO, International Federation of Gynecology and Obstetrics.

### 2.2 Brachytherapy procedure

#### 2.2.1 Patient preparation and imaging

Before brachytherapy, patients were instructed to empty their bladder and rectum. An indwelling Foley catheter was then placed with the patient in the lithotomy position. The Fletcher Williamson Asia Pacific applicator set was inserted based on pre‐brachytherapy diagnostic computed tomography (CT) or magnetic resonance imaging (MRI) and gynecological examination. When clinically indicated, interstitial needles were used to optimize target dose coverage. For treatment planning, an initial planning CT scan with a slice thickness of 3 mm was acquired on a CT simulator (Philips Brilliance Big Bore, Version 3.5.4) from the iliac crest to the ischial tuberosity with the applicator in situ. No slice thickness reduction or secondary image reconstruction was applied. When rectal gas was detected on the initial planning CT scan, evacuation was performed using a disposable enema wash package. The enema tube was inserted 15–30 cm into the rectum via the anus and removed after gas evacuation. A repeat CT scan was subsequently obtained using the same slice thickness (3 mm) as the initial planning CT and was used for final treatment planning. The indwelling Foley catheter remained clamped during the interval between the two CT acquisitions to maintain bladder filling. All patients tolerated the procedure without reported discomfort.

#### 2.2.2 Target and OAR delineation

CT images acquired before and after gas evacuation were imported into the Oncentra Brachy planning system (Elekta, Version 4.6.0). The high‐risk clinical target volume (HR‐CTV) and OARs were delineated on both image sets by a single experienced radiation oncologist in accordance with the ICRU Report No. 89 and the IBS‐GEC ESTRO‐ABS guidelines.^17^ The HR‐CTV encompassed the entire cervix and residual gross tumor. OARs included the bladder, rectum, sigmoid, and small intestine. The bladder was delineated from the dome to the proximal urethra, encompassing all identifiable bladder wall structures. The rectum was delineated from the superior margin of the anal canal to the rectosigmoid junction, following the outer rectal wall. The sigmoid was delineated from the rectosigmoid junction to the level at which the uterine body was no longer visible. The small intestine was delineated to include the intestinal loops and mesentery, extending to the superior margin of the uterine body. In routine clinical practice, final treatment planning was performed based on post‐gas evacuation images.

#### 2.2.3 Treatment planning and optimization

Brachytherapy planning was performed by a single experienced medical physicist on pre‐ and post‐evacuation CT image sets to ensure accurate and consistent applicator reconstruction. All plans were prescribed 600 cGy to the HR‐CTV. Importantly, each plan was independently reoptimized. Optimal treatment parameters were achieved through graphical optimization or manual adjustment of source dwell patterns (e.g., times and positions). Dose constraints recommended by the EMBRACE II study were followed.^18^ Treatments were delivered using the Flexitron HDR afterloader (Elekta AB, Stockholm, Sweden) with an Ir‐192 source.

### 2.3 Dosimetric parameters

DVH parameters before and after rectal gas evacuation were extracted, including HR‐CTV D_90%_ and *D*_98%_ (doses received by at least 90% and 98% of the HR‐CTV, respectively); OARs *D*_0.1cc_, *D*_1cc_, *D*_2cc_, and *D*_5cc_ (minimum doses to the most irradiated 0.1 cm^3^, 1 cm^3^, 2 cm^3^, and 5 cm^3^ of each OAR, respectively); and *D*_max_, *D*_mean_, as well as rectal and bladder volumes. The time interval between the two CT scans (Time_interval) was also recorded.

### 2.4 Factors associated with post‐evacuation bladder volume

Univariate and multivariate regression models were constructed to assess the effects of age, bladder volume before gas evacuation (Pre_volume), and the time interval between the two scans (Time_interval) on the bladder volume after gas evacuation (Post_volume). The multivariate regression model was defined as follows:

$$\begin{array}{ccc} \mathrm{Post}\_\mathrm{volume} & = & \beta_{0}+\beta_{1}\;\mathrm{Age}+\beta_{2}\;\mathrm{Pre}\_\mathrm{volume}\\ & & +\;\beta_{3}\;\mathrm{Time}\_\mathrm{interval}+\varepsilon \end{array}$$

where $\beta_{0}$ is the intercept, $\beta_{1}$ to $\beta_{3}$ are the regression coefficients for the independent variables, and ε is the error term.

### 2.5 Coordinate system definition and applicator displacement assessment

A bone‐based coordinate system was established individually for each patient using pelvic landmarks (e.g., the pubic symphysis and bilateral anterior superior iliac spines).^19^, ^20^ This system ensured stable and reproducible quantification of applicator displacement within the same patient and treatment fraction. Different patients, as well as different fractions within the same patient, were analyzed using independent coordinate systems. The procedure was performed as follows. First, the coordinates of pelvic landmarks on CT images before and after gas evacuation were recorded. Next, rigid registration of these landmarks was performed to minimize global positional deviations caused by patient repositioning or interscan variations. A local coordinate system was then established after gas evacuation, with the *x*‐, *y*‐, *z*‐axes oriented in the left–right, cranial–caudal, and ventral–dorsal directions, respectively. The anatomical position of the sacrum before and after gas evacuation was used to validate the accuracy of coordinate system registration, ensuring that deviations remained within an acceptable threshold (< 2–3 mm).^21^

As shown in Table 1, applicators used in this study were classified into five types: types 1–3 included a tandem, whereas types 4–5 did not. Accordingly, displacement assessment was tailored to the applicator configuration. For applicators containing a tandem (types 1–3), the “tip” was defined as the distal end of the tandem. In addition, the midpoint of the cervical stopper was recorded both before and after gas evacuation. For applicators without a tandem (types 4–5), the “tip” was defined as the distal end of the applicator component (e.g., vaginal cylinder or interstitial needle) that was closest to the geometric center of the HR‐CTV. Coordinates of these key positions were evaluated within the registered coordinate system to accurately quantify applicator displacement before and after gas evacuation.

### 2.6 Statistical analysis

Statistical analyses were conducted using SPSS version 26.0. Categorical variables were presented as counts (*n*) and percentages (%). Continuous variables were presented as mean ± standard deviation. Differences in DVH parameters before and after gas evacuation were analyzed using the Wilcoxon signed‐rank test, given the paired design and the non‐normal distribution of the data. These parameters were predefined and limited in number, and the analyses focused on planned within‐subject comparisons of primary endpoints. Therefore, formal adjustment for multiple comparisons (e.g., Bonferroni or Holm correction) was not applied. Due to the retrospective nature of the study, no a priori sample size or power calculation was performed, and the sample size was determined by the number of eligible patients meeting the predefined inclusion criteria during the study period. Model validity was assessed by evaluating multicollinearity using variance inflation factors (VIF). A *p*‐value less than 0.05 was considered statistically significant.

## 3 RESULTS

A total of 21 patients were included in the study, and their baseline characteristics are shown in Table 1. Rectal gas was predominantly observed during the early fractions of brachytherapy (the first and second fractions). Gas evacuation was required in 25 treatment fractions, resulting in 50 treatment plans (25 before and 25 after gas evacuation). The tandem was employed in 84% of all treatment procedures, with the specific applicator configurations detailed in Table 1. The mean time interval between the pre‐ and post‐gas evacuation CT scans was 453.56 ± 220.09 seconds.

### 3.1 Volumetric and dosimetric changes before and after rectal gas evacuation

**FIGURE 2:**
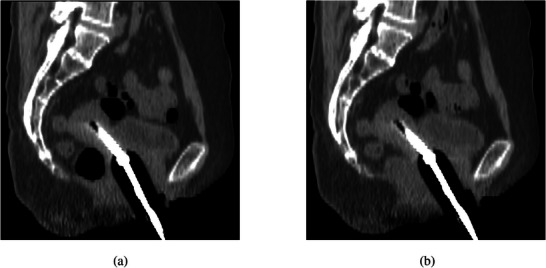
Representative CT images before and after rectal gas evacuation (a): pre‐evacuation; (b): post‐evacuation).

**FIGURE 3:**
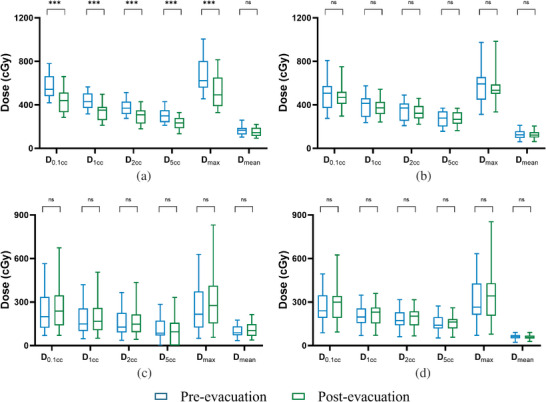
Box‐and‐whisker plots of dosimetric parameters (a): rectum; (b): bladder; (c): sigmoid; (d): small intestine). ***, *P* < 0.001; ns, *P* > 0.05.

Figure 2 illustrates the comparison of CT images before and after rectal gas evacuation. HR‐CTV volume showed no significant difference before and after gas evacuation (30.88 ± 20.07 vs. 30.92 ± 20.09 cm^3^, *P* > 0.05). Rectal volume decreased significantly by 25.11 ± 16.26 cm^3^ (62.67 ± 23.26 vs. 37.56 ± 13.10 cm^3^), whereas bladder volume increased significantly by 16.22 ± 17.09 cm^3^ (88.96 ± 26.68 vs. 105.18 ± 37.21 cm^3^). No significant differences were observed in HR‐CTV *D*_90%_ or *D*_98%_ (*D*_90%_: 600.46 ± 0.22 vs. 600.45 ± 0.20 cGy; *D*_98%_: 478.51 ± 22.06 vs. 473.97 ± 38.38 cGy). In the rectum, *D*_0.1cc_, *D*_1cc_, *D*_2cc_, *D*_5cc_, and *D*_max_ decreased significantly (*P* < 0.001), while the reduction in *D*_mean_ was not significant (*P* = 0.109). For the bladder, all DVH parameters except *D*_5cc_ decreased slightly without statistical significance (*P* > 0.05). *D*_5cc_ for the sigmoid colon and *D*_mean_ for the small intestine decreased, whereas other parameters exhibited minor increases, all without statistical significance (Figure 3).

### 3.2 Predictors of post‐evacuation bladder volume

Univariate regression analysis revealed a significantly positive correlation between the bladder volume before gas evacuation (Pre_volume) and the bladder volume after gas evacuation (Post_volume) (*P* < 0.001). No statistically significant correlations were observed for the other variables, including age (Age) and the time interval between the two CT scans (Time_interval). The multivariate regression analysis confirmed that Pre_volume was the sole significant predictor of Post_volume ($\beta_{2}$ = 1.304, *P* < 0.001). For every 1 cm^3^ increase in Pre_volume, Post_volume increased on average by 1.304 cm^3^. Age ($\beta_{1}$= 0.108, *P* = 0.770) and Time_interval ($\beta_{3}$= 0.017, *P* = 0.323) were not significant independent predictors of Post_volume (Table 2). The multivariate regression model was expressed as Post_volume = –24.894 + 0.108 Age + 1.304 Pre_volume + 0.017 Time_interval. The model demonstrated a high goodness of fit (*R* ^2^ = 0.834, adjusted *R* ^2^ = 0.81) and was statistically significant [*F*(3,21) = 35.1, *P* < 0.001]. All predictors showed VIF values below 5, indicating no significant multicollinearity.

**TABLE 2:** Linear regression analysis identifying predictors of bladder volume after rectal gas evacuation.

	Univariate analysis		Multivariate analysis	
	Coefficient (95% CI)	*P* value	Coefficient (95% CI)	*P* value
Age	0.167 (–1.477, 1.810)	0.836	0.108 (–0.651, 0.866)	0.770
Pre_volume	1.267 (1.016, 1.518)	<0.001	1.304 (1.035, 1.573)	<0.001
Time_interval	−0.029 (–0.101, 0.043)	0.416	0.017 (–0.018, 0.052)	0.323

### 3.3 Applicator displacement following rectal gas evacuation

**FIGURE 4:**
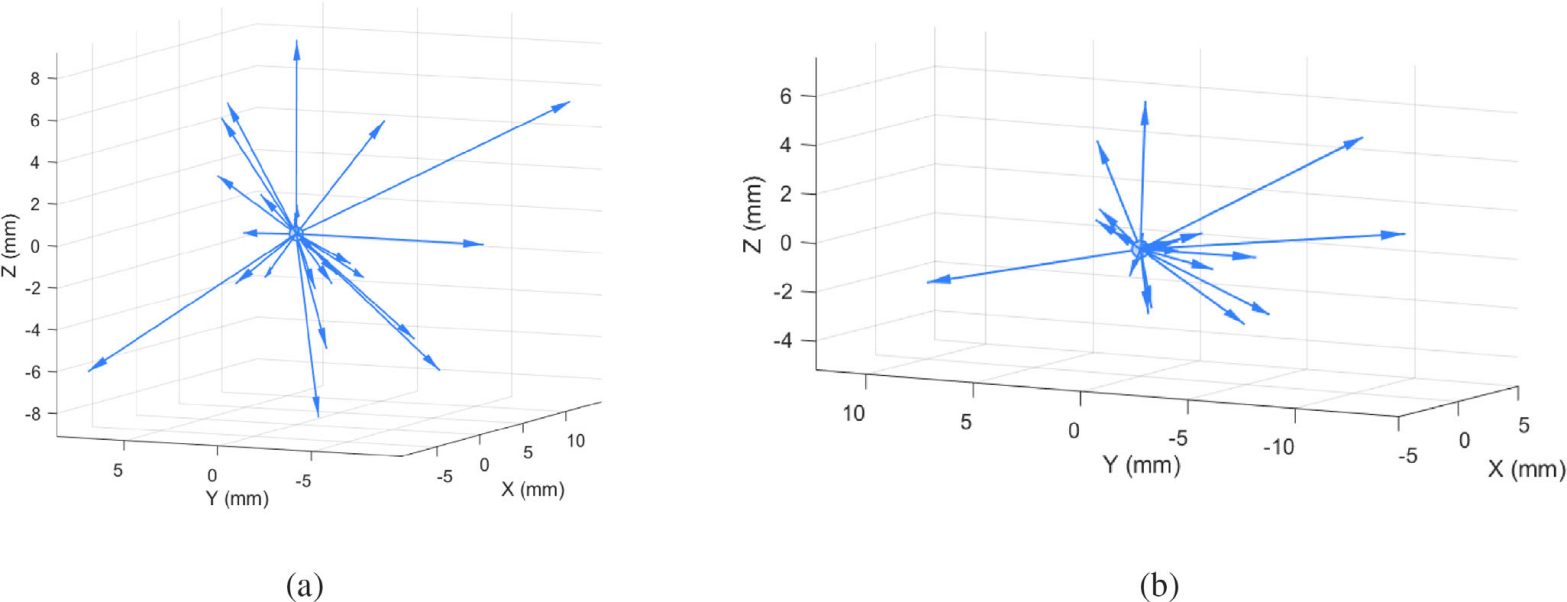
Three‐dimensional displacement vectors of the applicator (a): applicator tip; (b): cervical stopper).

After coordinate system registration, the sacral displacement error between the pre‐ and post‐gas evacuation CT scans ranged from 0.51 to 2.84 mm, with a mean of 1.50 ± 0.68 mm. These values were within the target registration error (TRE) range (< 2–3 mm) recommended in the TG‐132 report.^21^ The displacement of the applicator tip ranged from 0.99 to 15.44 mm, with a mean of 5.86 ± 3.64 mm. The displacement in the ventral–dorsal direction was greater than that in the left–right and cranial–caudal directions, predominantly in the dorsal direction (16/25, 64%). In the left–right direction, displacement occurred more frequently toward the left (17/25, 68%), whereas in the cranial–caudal direction, displacement was mainly caudal (14/25, 56%). For the cervical stopper, displacement ranged from 0.67 to 12.26 mm, with a mean of 4.23 ± 3.30 mm. Cranial–caudal displacement was greater than that in the left–right and ventral–dorsal directions, with caudal movement predominating (14/21, 66.67%). In the left–right direction, displacement was more often toward the left (13/21, 61.90%), while in the ventral–dorsal direction, displacement was mainly ventral (12/21, 57.14%). Detailed displacement values for the applicator tip and cervical stopper in each direction are provided in Table 3, and the corresponding three‐dimensional displacement vectors are illustrated in Figure 4.

**TABLE 3:** Displacement magnitudes of applicator tip and cervical stopper in the left–right (LR), cranial–caudal (CC), and ventral–dorsal (VD) directions.

		Applicator tip			Cervical stopper		
		LR	CC	VD	LR	CC	VD
Magnitude of displacement (mm)	Mean	2.43	3.17	3.42	1.45	3.04	1.69
	SD	2.39	2.70	2.67	1.20	3.24	1.68
	Range	0.06–10.53	0.10–9.79	0.31–8.85	0.04–4.02	0.07–10.93	0.24–5.76

## 4 DISCUSSION

In this retrospective study, we evaluated the effects of rectal gas evacuation on radiation doses to the HR‐CTV and OARs, organ volume, and applicator position in cervical cancer brachytherapy. Gas evacuation significantly reduced rectal volume and rectal dose. However, it was also accompanied by bladder volume changes and applicator displacement, which warrant careful attention in clinical practice.

This study demonstrated that gas evacuation significantly reduced rectal volume (from 62.67 ± 23.26 to 37.56 ± 13.10 cm^3^) and rectal DVH parameters (*D*_0.1cc_, *D*_1cc_, *D*_2cc_, *D*_5cc_, and *D*_max_) (Figure 3). These results align with the observations of Feree et al.^15^ and Vergalasova et al.^16^ According to the rectal complication model based on the EMBRACE study, the complication risk decreased from 9.5% to 6.9% after evacuation, underscoring the potential clinical value of this procedure in reducing rectal injury.^15^

This study showed that bladder volume increased significantly after gas evacuation, whereas bladder DVH parameters (*D*_0.1cc_, *D*_1cc_, *D*_2cc_, *D*_max_, and *D*_mean_) were slightly reduced. These findings are consistent with those reported by Feree et al.^15^ and Vergalasova et al.^16^ However, Sabater et al. observed an increase in bladder dose after gas evacuation, a discrepancy potentially attributable to the fact that all patients in that study had undergone prior surgery.^22^ Previous studies primarily focused on changes in bladder dose before and after gas evacuation, without considering changes in bladder volume. Evidence regarding the relationship between bladder volume and radiation dose remains conflicting: most researchers suggest that increased bladder filling reduces bladder radiation dose,^23^, ^24^ while others have reported an increase.^25^, ^26^ In brachytherapy, OAR dose is strongly determined by spatial geometry relative to the applicator. Changes in bladder volume can alter both bladder morphology and its position relative to the applicator, concurrently with applicator displacement that can be induced by rectal gas evacuation. Clinically, bladder irrigation is typically performed twice to maintain consistency: before the planning CT scan and again immediately prior to treatment delivery. In the present study, the average time interval between CT scans before and after gas evacuation was only 453.56 ± 220.09 seconds. During this brief period, only gas evacuation was performed, with no additional bladder management. Nevertheless, the regression model established in the present study showed that final bladder volume was closely related to baseline volume. Based on these findings, we recommend performing bladder irrigation after gas evacuation to maintain bladder volume consistency and minimize its impact on dosimetry.

This study is the first to quantitatively assess displacements of the applicator tip and cervical stopper in a three‐dimensional coordinate system before and after gas evacuation. The mean displacement of the applicator tip was 5.86 ± 3.64 mm, with larger movements observed along the cranial–caudal direction and ventral–dorsal direction than in the left–right direction. The position of the cervical stopper was relatively stable due to packing, with a smaller overall displacement (4.23 ± 3.30 mm) compared with the applicator tip. Its movement was more pronounced in the cranial–caudal direction, predominantly toward the caudal direction (14/21, 66.67%). This finding is consistent with previous studies on inter‐fraction uterine and cervical motion, which reported that uterine displacement was larger than the cervical displacement and was more pronounced in the ventral–dorsal and cranial–caudal directions.^27^ Despite applicator fixation, intrapelvic pressure and structural changes caused by rectal gas evacuation may still induce multi‐directional displacements, including left–right, cranial–caudal, and ventral–dorsal shifts. Due to the steep dose gradients in HDR brachytherapy, even millimeter‐level deviations can meaningfully alter dose distribution. Sandun et al. simulated caudal applicator displacement while maintaining dwell times and showed that shifts exceeding 5 mm led to clinically meaningful reductions in HR‐CTV D_90%_, accompanied by an increase in rectal dose.^28^ In our study, although the mean tip displacement after evacuation was 5.86 mm, all post‐evacuation plans were individually reoptimized, restoring HR‐CTV D_90%_ to the prescribed dose level. Therefore, applicator displacement on the order of 5 mm may be considered a clinically relevant threshold, and image‐guided reassessment with timely replanning is recommended whenever displacement approaches or exceeds this value.

This study has several limitations. First, it was a single‐center, retrospective study with a relatively small sample size, which may limit the generalizability of the findings. Validation in multicenter studies with larger cohorts is warranted to confirm the applicability of these results. Second, the use of various applicator types among patients may have interfered with the assessment of certain dosimetric parameters when evaluating applicator displacement.

## 5 CONCLUSIONS

Rectal gas evacuation results in a statistically significant and clinically relevant reduction in rectal volume and rectal dose, underscoring its importance as a routine clinical procedure. However, as it may induce millimeter‐level applicator displacement with clinically measurable dosimetric consequences, careful monitoring is warranted, with post‐evacuation replanning or, if necessary, applicator adjustment.

## AUTHOR CONTRIBUTIONS

**Haiyan Wu**: Conceptualization, methodology, formal analysis, writing—original draft, writing—review and editing. **Chengdian He and Mei Liu**: Data Curation, writing—review and editing. **Xiujuan Zhao**: Conceptualization, supervision, funding acquisition, writing—review and editing. All authors read and approved the final manuscript.

## FUNDING INFORMATION

This work was supported by Chongqing Technology Innovation and Application Development Special Project‐Key project (No. CSTB2022TIAD‐KPX0152), Chongqing Innovative Medical Device Application Demonstration Project (No. CQEIC2024MDAD‐057), and Scientific and Technological Research Program of Chongqing Municipal Education Commission (No. KJQN202300132).

## CONFLICT OF INTEREST STATEMENT

The authors have no relevant conflicts of interest to disclose.

## Data Availability

The data will be shared on reasonable request to the corresponding author.
